# Minimal change disease in graft versus host disease: a podocyte response to the graft? 

**DOI:** 10.5414/CN107420

**Published:** 2013-09-25

**Authors:** Janna Huskey, Chris Rivard, Han Myint, Scott Lucia, Maxwell Smith, Michiko Shimada, Takuji Ishimoto, Carlos Araya, Eduardo H. Garin, Richard J. Johnson

**Affiliations:** 1 Division of Renal Disease and Hypertension,; 2 Division of Hematology and Oncology, and; 3 Department of Pathology, University of Colorado, Denver, CO,; 4 Division of Pathology Mayo Clinic Phoenix, Phoenix, AZ, and; 5 Division of Pediatric Nephrology, University of Florida, Gainesville, FL, USA

**Keywords:** graft versus host disease, minimal change disease, urinary CD80

## Abstract

Nephrotic syndrome is a rare complication of hematopoietic cell transplantation. It has been suggested that nephrotic syndrome may represent a limited form of graft-versus-host disease although the pathological link between these two entities remains unclear. In this paper, we report a case of a 61-year-old female who underwent nonmyeloablative allogenic stem cell transplantation for T-cell prolymphocytic leukemia and subsequently developed biopsy proven minimal change disease shortly after cessation of her immunosuppression therapy. Urinary CD80 was markedly elevated during active disease and disappeared following corticosteroid-induced remission. We hypothesize that alloreactive donor T cells target the kidney and induce podocyte expression of CD80 that results in proteinuria from limited ‘graft versus host’ disease.

## Introduction 

Hematopoietic cell transplantation (HCT) is an established treatment for various hematologic diseases. Acute kidney injury (AKI) complicates 22 – 76% of patients with HCT depending on the type of transplant received [1]. Radiation nephropathy, calcineurin inhibitor toxicity, transplant-associated thrombotic microangiopathy, and chronic graft-vs.-host disease (GVHD) contribute to chronic kidney disease and develops in ~ 17% of patients [2, 3]. The development of nephrotic syndrome after HCT is unusual [4, 5, 6, 7, 8, 9]. Whilst a variety of glomerular diseases have been noted, the most commonly observed nephrotic syndrome after HCT is membranous nephropathy followed by minimal change disease (MCD) [6, 7, 8, 9, 10, 11, 12]. It has been suggested that a link between chronic GVHD and glomerular disease exists, however the pathogenesis remains unclear [7]. 

T cells play a crucial role in the pathogenesis of both GVHD and MCD [13, 14, 15, 16]. In chronic GVHD, alloreactivity, and possible autoreactivity, of donor T cells leads to cytokine stimulation, B cell activation, autoantibody production, and organ damage [17]. MCD has been commonly thought of as a T cell disorder and has recently been proposed to be a “two hit” podocyte immune disorder [18]. First, an immunologic stimulus results in podocyte expression of CD80, a transmembrane protein expressed on antigen presenting cells (APCs) involved in costimulation and T-cell activation [19]. This leads to rearrangement of the actin cytoskeleton, alterations in the slit diaphragm, and proteinuria [19]. The “second hit” results from dysfunctional T regulatory cells (Tregs) which are unable to inactivate the immunologic stimulus that triggers the increased podocyte CD80 expression leading to persistent nephrotic syndrome [18]. We present a case of biopsy proven GVHD associated MCD, as a sole manifestation of chronic GVHD, in which we hypothesize that the alloreactive donor T cells serve as the immunologic stimulus that leads to podocyte expression of CD80 and nephrotic syndrome. 

## Case

A 61-year-old female with T-cell prolymphocytic leukemia underwent an allogenic stem cell transplant from her sister who served as a 10 out of 10 HLA-matched sibling donor in August 2008. In 2005, she presented with leukocytosis and bone marrow biopsy revealed T-cell prolymphocytic leukemia for which she received alemtuzumab, a humanized anti-CD52 antibody, and subsequently went into remission. Repeat bone marrow biopsy in 2008 revealed disease recurrence at which time a reduced intensity conditioned allogenic stem cell transplant was performed (conditioned with fludarebine and melphalan) without any major complications. Her (GVHD) prophylaxis was tacrolimus and methotrexate, as per our institutional protocol. Day 100 after transplant, her tacrolimus dose was lowered and within a few weeks, she developed a rash that was consistent with a clinical diagnosis of chronic GVHD. Prednisone (1 mg/kg) was initiated and tacrolimus was increased. As her rash improved, her immunosuppression was gradually tapered over 9 months. 

**Figure 1.**
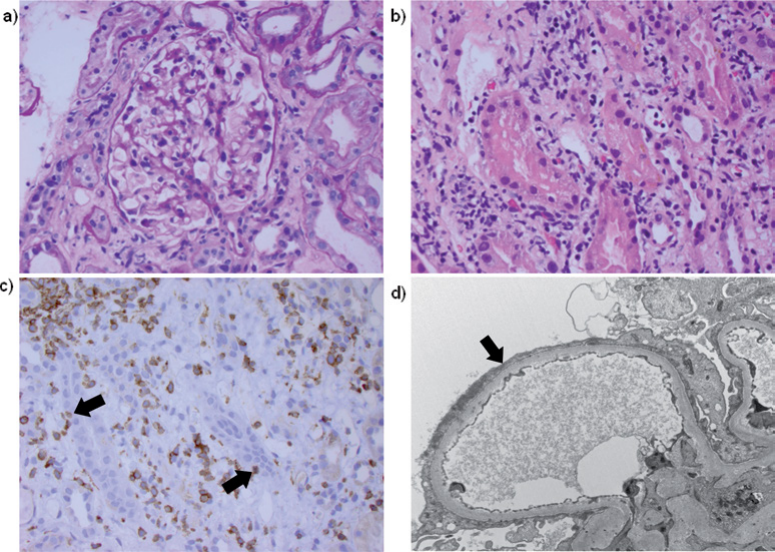
Light and EM. a: Normal glomerulus showing open capillary loops, thin delicate basement membranes, and no significant hypercellularity. No segmental scars were identified (400×, PAS stain). b: Interstitial inflammation and tubular damage (400×, H&E stain). c: CD3 predominant T-cell interstitial inflammatory cell infiltrate with areas of tubulitis (arrow) (400×, CD3 immunohistochemical stain). d: Severe foot process effacement (podocyte fusion) (arrow) (Electron Microscopy).

Three weeks after stopping all immunosuppression, she developed nausea, fatigue, and edema. She was noted to have new onset nephrotic syndrome manifested by anasarca, hypoalbuminemia (1.8 mg/dl), hyperlipidemia (total cholesterol 307 mg/dl with LDL 185 mg/dl, triglycerides 396 mg/dl), proteinuria (19.9 g/day) and an elevated serum Cr of 2.4 mg/dl (baseline 0.9 – 1.1 since May 2008). Extensive serologic workup was negative. Renal biopsy revealed interstitial inflammation, tubulitis, tubular atrophy and normal glomeruli by light microscopy with diffuse foot process effacement on electron microscopy (Figure 1). JC virus, Epstein-Bar virus, and cytomegalovirus immunohistochemical stains were negative. Urine eosinophils, serum and urine BK virus, serum enterovirus, serum HHV-6, and serum HHV-8 were negative. Short tandem repeat chimerism studies showed that ~ 32% of the DNA material in the biopsy specimen originated from donor cells [5]. Immunohistochemical stains showed a predominantly CD3 positive T-cell infiltrate with increased CD8+ T cells. Repeat bone marrow biopsy was negative for disease recurrence and she had full donor chimerism of all fractions tested including CD3, CD33, and CD56 on her bone marrow. The presence of donor T cells in her kidney, lack of nephrotoxic medications such as non-steroid anti-inflammatory drugs, and a bone marrow biopsy indicating full donor chimerism without signs of disease recurrence lead to a diagnosis of chronic GVHD in the kidney manifested as MCD. 

The patient received prednisone 2 mg/kg/d, furosemide, and statin therapy and was discharged from the hospital with a Cr of 2.9 mg/dl (peaked at 3.2 mg/dl). Within 8 weeks, her proteinuria had improved to 1.3 g/d and her Cr decreased to 0.9 – 1.1 mg/dl. The prednisone was tapered over 4 months and she went into complete renal remission with < 500 mg protein in a 24-h urine collection. Nevertheless, her rash returned and mycophenolate mofetil was added to her regimen of prednisone 15 mg/d for GVHD. Over the next several months, her proteinuria improved and the mycophenolate mofetil was discontinued, and in July 2010 her prednisone was decreased to 5 mg every other day. In September 2010, her nephrotic syndrome returned with proteinuria (9.5 g/d), anasarca (11.3 kg weight gain), hyperlipidemia (total cholesterol 332 mg/dl, LDL 229 mg/dl, triglycerides 270 mg/dl), and a Cr of 1.9 mg/dl. Her prednisone was increased to 1 mg/kg and furosemide and statin therapy were restarted. Repeat chimerism of the peripheral blood confirmed full donor chimerism on all 3 fractions tested. By December 2010, her proteinuria fell to 500 mg/d and her renal function returned to baseline. She was treated with a slow prednisone taper and continues to remain in remission with undetectable proteinuria on prednisone 10 mg daily. 

## Urinary detection of CD80 

**Figure 2.**
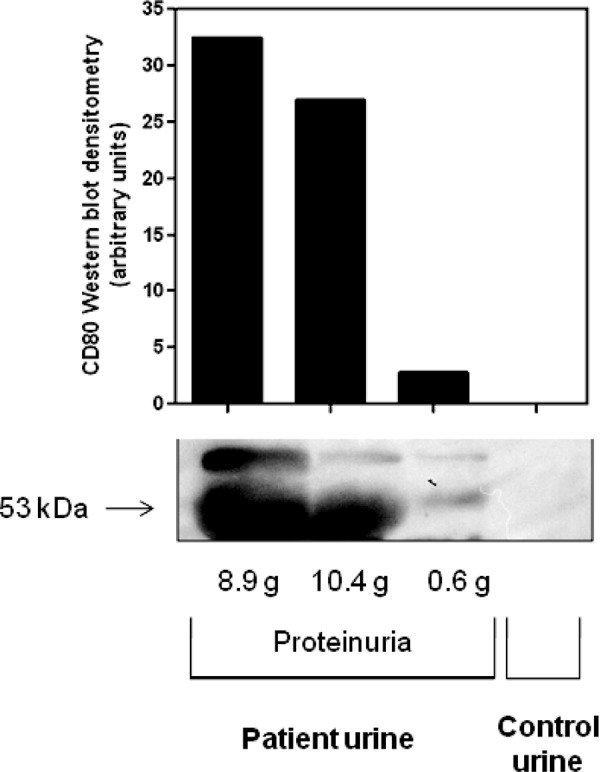
Western blot of urinary CD80: Urine samples were warmed to room temperature, vortexed, and centrifuged at 8,000 g for 5 min. Total urine protein was determined by the bicinchoninic acid (BCA) protein assay (Pierce, Rockford, IL, USA). 30 µg total protein was loaded per lane in a 4 – 20% precast Criterion polyacrylamide gel (Bio-Rad, #345-0033, Hercules, CA, USA). Proteins were transferred to an Immobilon transfer membrane (Millipore, Billerica, MA, USA), blocked for 1 hour in 5% milk and incubated overnight at 4 °C with anti-CD80 antibody (R&D Systems, #AF-140, Minneapolis, MN, USA). The membrane was washed in Tween-Tris Buffered Saline (TTBS) and revealed using an antimouse horse radish peroxidase (HRP) linked secondary antibody (Cell Signalin, #7076S, Danvers, MA, USA) and Immun-Star reagent (Bio-Rad). The membrane was exposed to film and images evaluated for densitometry using 1D Image Software (Kodak Digital Science, Rochester, NY, USA). Patient urine samples collected during MCD relapse (8.9 g, 10.4 g of proteinuria) and during partial remission (0.6 g proteinuria) were compared to a healthy control urine sample. All samples were frozen following collection at –20 °C until analyzed.

We have reported that children with idiopathic MCD express CD80 in their podocytes in association with high levels of the membrane-bound (53 Kd) CD80 protein in their urine during relapse [20, 21]. To determine if a similar pattern was occurring in this patient, and following consent of the patient and approval by the University Human Subjects Committee, urine was collected both during relapse and following remission and Western blotting for CD80 was performed according to standard procedures [21]. As shown in Figure 2, a marked increased urinary excretion of membrane-bound CD80 (53 Kd) was observed during relapse. Excretion was dramatically lower but still slightly detectable when her proteinuria improved to ~ 600 mg/day and was absent in a healthy control. Glomerular staining for CD80 was not attempted due to lack of frozen tissue that is required for immunostaining with the anti-CD80 antibody. 

## Discussion 

MCD is classically considered a T-cell disorder [13]. A breakthrough in our understanding of this condition was provided by Reiser et al. [19] who discovered that CD80, a receptor normally expressed on dendritic cells, can be induced in podocytes. The injection of lipopolysaccharide (LPS) in mice resulted in CD80 expression in the podocytes with foot process effacement and proteinuria and this was not observed when LPS was injected into CD80 knockout mice [19]. As the histologic findings were similar to MCD, Reiser and Mundel [22] suggested that this might underlie the pathogenesis of this condition. Our group subsequently documented that urinary CD80 excretion is high in corticosteroid responsive MCD and appears to be relatively specific to this disease entity [20, 21]. Urinary CD80 values are significantly higher in patients with relapsing MCD when compared with patients with MCD in remission, other glomerular diseases such as focal segmental glomerulosclerosis (FSGS) or membranous nephropathy, and healthy controls [20, 21]. We were able to identify CD80 in the podocytes of a limited number of renal biopsies of relapsing MCD, and to show that the CD80 present in the urine was the membrane-bound (53Kd MW) form and not circulating soluble CD80 (23 Kd MW) [21]. Here we report a case of corticosteroid-sensitive MCD associated with GVHD that is also associated with increased urinary CD80 expression. While we were not able to determine if CD80 was expressed in the glomerulus, the observation that the urinary CD80 in this patient was membrane bound (53 Kd MW) and not the circulating form (23 Kd MW) strongly suggests that the pathogenesis of the proteinuria in this case is similar to MCD and is a form of CD80 podocytopathy. 

In mice, the induction of proteinuria by LPS results in only transient CD80 expression and transient proteinuria [19]. Since CD80 can be induced in podocytes by various Toll-like receptor ligands and cytokines, we have hypothesized that this might explain the transient proteinuria that is occasionally observed in febrile patients [23]. However, persistent CD80 expression might require the continuous exposure of a cytokine because of impaired Tregs [24] or the inability of the host podocyte to censor the CD80 response [23]. 

In this case, GVHD provides a mechanism for continuous immune stimulation by the grafted cells to the host. T cells from the graft have been identified in the interstitium of subjects with MCD following allogeneic stem cell transplantation [5]. Furthermore, a reduced Treg population has been associated with chronic GVHD [25]. Finally, a recent study found a reduction in Tregs in the kidney biopsies of subjects with nephrotic syndrome (of various causes) following allogeneic stem cell transplantation [26]. This reduction in Tregs might facilitate the clonal expansion of host reactive T cells (memory function primed to antigens on the podocyte) that could be responsible for the disease. Since the podocyte can function as a dendritic cell and can express CD80 in the setting of an immunologic stimulus, such as T cell directed response to antigens on the podocyte, this could provide a mechanism for explaining the pathogenesis of proteinuria in this condition. It also remains possible that either methotrexate or tacrolimus may have a role in the proteinuria, although neither agents are associated with the development of MCD in the nontransplant setting [27, 28]. Further studies are needed to better identify the cellular mechanisms driving the CD80 response in this disease. 

## Funding 

Supported by NIH grant DK-80764.
